# Computing Endolymph Hydrodynamics During Head Impulse Test on Normal and Hydropic Vestibular Labyrinth Models

**DOI:** 10.3389/fneur.2020.00289

**Published:** 2020-04-21

**Authors:** Jorge Rey-Martinez, Xabier Altuna, Kai Cheng, Ann M. Burgess, Ian S. Curthoys

**Affiliations:** ^1^Neurotology Unit, ENT Department, Hospital Universitario Donostia, San Sebastián - Donostia, Spain; ^2^Vestibular Research Laboratory, School of Psychology, The University of Sydney, Sydney, NSW, Australia

**Keywords:** menière disease, endolymphatic hydrops, vHIT, VOR, clinical sign, enhanced eye velocity, CFD

## Abstract

**Hypothesis:** Build a biologic geometry based computational model to test the hypothesis that, in some circumstances, endolymphatic hydrops can mechanically cause enhanced eye velocity responses during clinical conditions of the head impulse test.

**Background:** Some recent clinical and experimental findings had suggested that enhanced eye velocity responses measured with the video head impulse test could not only be caused by recording artifacts or central disfunction but also could be directly caused by the mechanical effect of endolymphatic hydrops on horizontal semicircular canal receptor.

**Methods:** Data from clinical video head impulse test was computed in three biologic-based geometry models governed by Navier-Stokes equations; six head impulses of incrementally increasing peak head velocity were computed in each one of the three different geometric models, depending on absence, canal or utricular hydrops.

**Results:** For all computed head impulses an increased endolymphatic pressure was measured at the ampullar region of the horizontal semicircular canal on both canal and utricular hydrops models. The mean of aVOR gain was 1.01 ± 0.008 for the no-hydrops model, 1.14 ± 0.010 for the canal hydrops model was, and 1.10 ± 0.007 for the utricular hydrops model.

**Conclusion:** The results of the physical computation models support-the hypothesis that in endolymphatic hydrops conditions, which are affecting horizontal semicircular canal and utricular region on moderate dilatations, the eye velocity responses output-by the aVOR will be enhanced by a 1.14 factor and aVOR gain values will be enhanced by over 1.1 for impulses to the right side.

## Introduction

The video head impulse test (vHIT) is a computer-quantified clinical test of semicircular canal function that has a wide clinical application for vestibular and central pathologies in which the angular vestibulo-ocular reflex (aVOR) is affected (1, 2). vHIT has two main quantified outputs that are used in clinical practice. The first is the quotient between eye and head velocity during the slow phase period (1) known as the gain of the aVOR (3) and the second is the timing, velocity and synchronicity of the saccadic eye responses produced during slow- or fast-phase periods (4).

About the gain of aVOR parameter it is also widely accepted that the presence of a lower value of aVOR gain—corresponding to situations where the slow-phase eye velocity is lower than head velocity most of the time—is a direct indicator of vestibular hypofunction (1), but recently published case reports have suggested that enhanced eye velocity responses during the head impulse test could be a quantified sign of endolymphatic hydrops (5). Enhanced aVOR responses are characterized by an enhanced eye velocity in response to head velocity. This enhanced eye velocity is reflected as a slight increase of the aVOR gain parameter, usually measured in a gain range between 1.1 and 1.3 (5). In this previously published case report we suggest that although enhanced eye velocity responses could be related with central vestibular disorders or vHIT goggle slippage due to an inadequate acquisition technique, there are some cases in which the most probable cause of the enhanced eye velocity responses is the endolymphatic hydrops.

Based on previous investigations (6), we suggest that the increase of volume and pressure on the horizontal semicircular canal will provoke an increased (transcupular) pressure on the cupula receptor that will also increase the vestibular afferent signal.

Because direct experimentation of this hypothesis on humans will cause loss of vestibular and auditive functions, researchers classically returned to experimental animal models to explore vestibular function tests (7) under different normal or pathologic biologic conditions. As an alternative to experiments, mathematical, and physical models supporting vestibular physiology do not cause any loss of biologic function, and they give strong evidence that modeled hypotheses are plausible from a physical and mathematical point of view. But these models are sometimes difficult to solve because of the complex mathematical methods required, making the physical-based predictions of the models very basic and imprecise.

Despite computer science has many outstanding figures who has significantly contributed to its development, it's probably since Alan Turing introduced the possibility of complex mathematical function computation by using recursive algorithms based on simple mathematical operations that could be automatically computed by a (theoretical) machine (8), that the use of computational models has grown significantly, with robust methods to develop realistic computer models to explain complex physical and biological phenomena. In the vestibular research field, these complex computational methods have recently been successfully used to model and predict vestibular caloric test responses in normal and endolymphatic hydrops on simplified anatomic models (9) or to reach a better understanding of the Tullio Phenomenon (10).

The main purpose of this research is to develop an anatomical model of the membranous vestibular labyrinth to compute and simulate endolymph hydrodynamics during real clinical head impulse test movement for models of both normal endolymph volume, and of endolymph volume increased by hydrops. With these models we will try to test the hypothesis that, in some circumstances, endolymphatic hydrops can mechanically cause enhanced eye velocity responses during head impulse testing in clinical conditions.

## Materials and Methods

### Development of Geometric Models of the Vestibular Organ for Normal and Hydropic Endolymph Volumes

The anatomic model used in this study was obtained from an intact *ex vivo* temporal bone using micro-computer tomography (microCT) scan; a Skyscan 1,172 (Bruker, DKSH Management Ltd, Zurich, Switzerland) microCT desktop scanner device was used in this study. Multiple images obtained using x-ray transmission were saved as uncompressed True Image File Format (TIFF) files.

**Figure 1:**
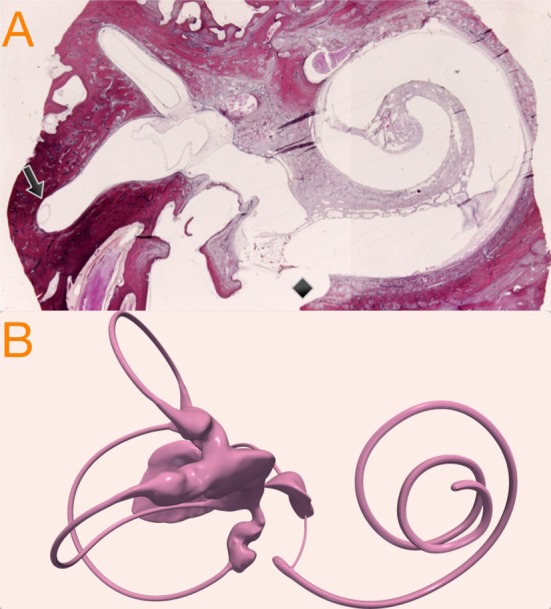
**(A)** Temporal bone histologic (hematoxylin-eosin) preparation showing cochlear and vestibular structures on a parasagittal plane. The black arrow indicates the relation of endolymph horizontal duct inside the osseous space. The black diamond shows post-mortem promontorial cochleostomy with endosteal layer injury and *scala tympani* traumatic penetration. **(B)** Micro-CT volumetric of the endolymphatic spaces of the membranous labyrinth reconstruction used in this study as original 3D model of the inner ear.

The TIFF image sequence of the temporal bone was then loaded, processed, combined and exported as a single file in Nearly Raw Raster Data (NRRD) file format. The NRRD file was loaded into 3D Slicer (https://www.slicer.org) reconstruction software where a final 3D reconstructed anatomic model surface of the endolymphatic spaces of the membranous labyrinth model was created as seen in Figure 1B. The reconstructed 3D model was finally exported as standard triangle language (STL) file format that was used in computations as the anatomic model, and was also used as the base model to develop the hydrops models.

To develop the hydrops model we considered as McGarvie et al. had previously published (11) that, in human anatomy, the horizontal semicircular duct can be expanded to six times its normal diameter, because of the relative space distribution between endolymph duct and semicircular bone canal (Figure 1A): these authors describe an endolymph duct of 0.23 mm inside a osseous canal of 1.53 mm.

**Figure 2:**
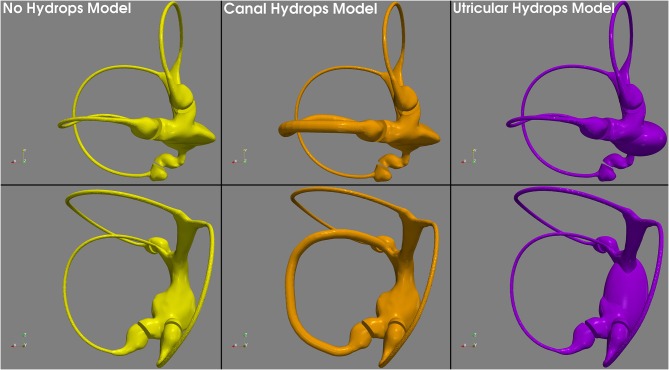
3D geometric models used in this research. The no hydrops model **(left)** obtained with conservative postprocessing from micro-CT scan, the canal hydrops model **(center)** with a uniform x3 horizontal endolymph duct dilatation, and the utricular hydrops model **(right)** with a similar to canal hydrops model expansion applied to the utricular region.

Based on this, because our 3D model duct has a diameter of 0.45 mm, we therefore allowed it to be uniformly increased x3 to reach a plausible 1.3 mm hydropic diameter, that still fit inside the osseous canal frame. Because utricular hydrops is not so well-defined in the literature, we decided to develop the hydrops model with a uniform expansion similar to the model of canal hydrops. Normal, canal hydrops and utricular hydrops are presented in Figure 2. The two hydrops models were designed using 3D computer design software Blender 2.8 (Blender—a 3D modeling and rendering package, Blender Foundation, Amsterdam, Netherlands; www.blender.org).

### Computer Fluid Dynamics Simulations

All developed 3D models were exported in STL file format to the computational fluid dynamics (CFD) software: fully licensed SimFlow 3.1 (SIMFLOW technologies, Warsaw, Poland; https://sim-flow.com) CFD software was used to perform the model simulations to explore the endolymph hydrodynamics during the head impulse test. Once the 3-D models were imported into SimFlow a high-resolution mesh was created using the STL geometric models, defining the outer surfaces of the volumetric model.

The next step in the computer simulation was to select the solver, the mathematic algorithms used to compute the fluid dynamics during head impulses. The transient state with dynamic mesh capability “*PimpleDyMFoam*” (OpenFOAM documentation, The OpenFOAM Foundation Ltd, London, England; https://www.openfoam.org) solver was used on parallel running for our head impulse test computations. “*PympleDyMFoam*” algorithm's governing equation is based on the Navier-Stokes equations (12) for incompressible fluids, considering the vestibular labyrinth as the rotating absolute velocity frame. According to the solver's algorithm developer (OpenFOAM documentation, The OpenFOAM Foundation Ltd, London, England; https://www.openfoam.org) these governing equations are:

$$\begin{array}{l} \{\begin{array}{c} \nabla •\left(\vec{u_{R}}⊗\vec{u_{I}}\right)+\vec{\Omega }\times \vec{u_{I}}=-\nabla \left(p/\rho \right)+\upsilon \nabla •\nabla \left(\vec{u_{I}}\right)\\ \nabla •\vec{u_{I}}\;=0 \end{array} \end{array}$$

Where $\vec{\Omega }$ is the acceleration of the rotating frame, I is notation for inertial movement, R is notation for rotating movement, $\vec{u}$ is (endolymph) velocity vector, ρ is the endolymph viscosity and *p* is the pressure. The ⊗ operator is used to denote the tensor product between velocity vectors.

For the external (vestibular labyrinth) rotational head velocity input vector we used head velocity data from a video head impulse test database at Royal Prince Alfred Hospital, Sydney. These impulses were all from the same participant and testing session and operator, and were selected to have values of peak head velocity ranging from 102.62°/s to 249.47°/s (Appendix Figure 1). The head impulses were recorded with a prototype ICS Impulse (GN Otometrics, Taastrup, Denmark) vHIT device with a sampling time of 0.004 s per frame, i.e., with an acquisition frequency of 250 Hz. The total duration recorded for each head impulse was 0.7 s.

The recorded head velocity data of the six impulses were lowpass-filtered and exported to the SimFlow software to be computed each one with so that each impulse could be applied to all of the three 3D models: normal, canal hydrops and utricular hydrops. For initial conditions endolymph and vestibular organ rotation velocities were set to 0 °/s, and the thermodynamic properties of endolymph were directly extrapolated from water thermodynamic properties: for the present research endolymph kinematic viscosity was 1 e-06 m^2^/s.

All computation step was recorded, step duration was set to 0.004 s and simulation total time was set to 0.7, to exactly agree with vHIT sampling frequency and impulse recording duration.

Accordingly, a total of eighteen head impulses were computed in three geometric biologic-based models governed by Navier-Stokes equations; six incremental peak velocity head impulses were computed in each one of the three different geometric models depending on absence, canal or utricular hydrops. CSV head impulse velocity data, STL vestibular geometric models and CFD configuration files for each geometric model are available at Appendix Datasheet 1–5.

### Computer Fluid Dynamics Simulations Postprocessing and Analysis

Computed head impulses were analyzed and postprocessed with Paraview 5.7.0 (Sandia National Laboratories, Albuquerque & Kitware Inc, New York, USA; https://www.paraview.org) software. Pressure and velocity 3D spaces and endolymph steam lines were the main outputs measured and visualized using Paraview; Appendix Videos 1–3 supplementary files shows the Paraview exported videos of the normal and canal hydrops models for the head impulse test VI. The main pressure outcome was measured by selecting as pressure receptors the middle and superior points of the internal wall of the ampullar region; the mean pressure measures over time were exported to be analyzed using MATLAB R2019B (The MathWorks, Inc, Natick, Massachusetts, USA; https://www.mathworks.com).

As was described by Grant and Curthoys, the ampullar crista receptor was considered as an highly overdamped pressure—accelerometer sensor that directly responds to endolymph displacement velocity (13) that is directly proportional to angular head velocity (14) and according to Ramat (15), the neural discharge of vestibular afferents for high frequency head movements, in the range of physiological movements, is also a transduction of head angular velocity.

But in our developed geometric model the cupula was modeled as a rigid wall and it was not possible to directly measure the cupula deflection that occurs on biological semicircular canal. As an approach, cupular volume displacement can be estimated from the variation over time of transcupular pressure, that was the main variable measured in our CFD model.

According to Squires et al. cupular volume displacement *V*_*c*_(*t*) is in relation to the applied pressure variation $\Delta P_{c}\left(t^{\prime }\right)$ by the equation (16):

$$\begin{array}{l} V_{c}\left(t\right)=\frac{1}{\gamma }\int_{-\infty }^{t}\Delta P_{c}\left(t^{\prime }\right)e^{\frac{-\left(t-t^{\prime }\right)}{\tau_{c}}}dt^{\prime } \end{array}$$

Where τ_*c*_, cupular time constant, is given by the equation (16):

$$\begin{array}{l} \tau_{c}=\;\frac{8\mu \beta_{d}R}{\pi b_{d}^{4}K} \end{array}$$

And γ is given by the equation (16):

$$\begin{array}{l} \gamma =\;\frac{8\mu \beta_{d}R}{\pi b_{d}^{4}} \end{array}$$

Where μ is the endolymph viscosity, β_*d*_ is the angle subtended by semicircular duct, *R* is the major radius of semicircular canal, *b*_*d*_ is the duct radius and *K* the cupular elastic constant.

Finally, head movement Ω_0_ is related with cupular volume displacement *V*_*c*_ by the expression, for human beings (16):

$$\begin{array}{l} \frac{\Omega_{0}}{V_{c}}\approx 5.6\times 10^{-2}\;\left(\frac{◦}{pL\;s}\right) \end{array}$$

With the computed cupular displacement *V*_*c*_ approximated from the measured Δ*P*_*c*_ on the CFD simulations for each (hydropic and not hydropic) models we obtained the theoretical head movement measured by the cupula in both normal and hydropic semicircular canal for each one of the six head impulses. Assuming that because the unique perturbation introduced in our models was the hydrops of the duct or utricle regions, keeping intact all the other structures from cupula to eye that participates on the aVOR response, we predicted the eye responses by applying a (normofunctional) aVOR gain of ~1 between eye and head responses. Using this approach, we predicted the final eye response based on the obtained head velocity by computing the measured Δ*P*_*c*_.

To calculate the gain of the aVOR response on the computer simulation results, the obtained eye velocity values for each head impulse computation area under curve value were divided by the area under the curve of the head impulse velocity data for the corresponding simulation. To measure the area under the curve from both real head velocity and computed eye velocity, the HITCal (4) open source calculus tool (https://github.com/bendermh/HITCal/releases) was used under a MATLAB computational environment.

## Results

Vestibular model computations with CFD software were successfully performed with a residuals level under 0.01 during the head impulse movement for all cases. None of the variables, grouped by geometric model, showed statistical differences from a normal distribution on Kolmogorov-Smirnov normality test (*p* > 0.5).

**Figure 3:**
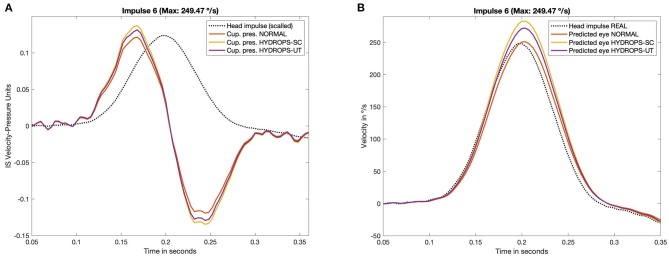
Measured pressure **(A)** on CFD simulation and predicted eye velocity responses **(B)** of the three hydrops models (non-hydrops, utricular hydrops and semicircular canal hydrops), for the head impulse with the highest peak velocity included in this study (249.47 °/s). The pressure and velocity plots obtained from all the others impulses included in this study are available in Appendix Figures 2, 3.

The main results of computations are presented in Table 1 and are also plotted in Figure 3. For measured ampullar pressure values (Table 1, Figure 3A and Appendix Figure 2) higher peak pressure was measured in canal hydrops model with a mean pressure between impulses of 0.107 ± 0.030 Pa, followed by utricular hydrops model with a mean pressure of 0.103 ± 0.028 Pa. Lowest peak pressure levels were measured in no-hydrops model with a mean pressure of 0.095 ± 0.027.

**Table 1:** Main outputs of the model's computation.

**Impulse**	**Hydrops model**	**Increment of ampular pressure (Pa)**	**Predicted eye velocity peak (^**°**^/s)**	**Head velocity peak (^**°**^/s)**
I	No Hydrops	0.054	102.33	102.62
II	No Hydrops	0.081	137.06	137.46
III	No Hydrops	0.089	141.22	140.50
IV	No Hydrops	0.100	173.22	173.81
V	No Hydrops	0.126	210.97	209.11
VI	No Hydrops	0.125	248.06	249.47
I	Canal Hydrops	0.061	115.79	102.62
II	Canal Hydrops	0.091	155.16	137.46
III	Canal Hydrops	0.101	159.74	140.50
IV	Canal Hydrops	0.113	195.53	173.81
V	Canal Hydrops	0.142	238.64	209.11
VI	Canal Hydrops	0.137	282.98	249.47
I	Utricular Hydrops	0.058	111.40	102.62
II	Utricular Hydrops	0.088	148.58	137.46
III	Utricular Hydrops	0.097	152.82	140.50
IV	Utricular Hydrops	0.109	184.43	173.81
V	Utricular Hydrops	0.136	229.01	209.11
VI	Utricular Hydrops	0.131	272.03	249.47

*Computationally measured increment of pressure on the internal wall of the ampulla, and predicted eye velocity values according to measured pressure levels for all the six head impulses on each three anatomical hydrops models*.

Predicted eye velocity peak values (Table 1, Figure 3B and Appendix Figure 3) were also highest in canal hydrops model, with a mean peak eye velocity of 191.355 ± 61.10 °/s, followed by peak velocity values from utricular hydrops model of 183.61 ± 58.79 °/s. Lowest peak eye velocity values were also measured in no-hydrops model with a mean velocity of 168.82 ± 53.51 °/s.

For all models highest peak pressure and peak eye velocity were measured on V and VI impulses (209.11 °/s and 249.47 °/s head peak velocity values, respectively) while lowest predicted peak eye velocity and peak pressure vales were measured on impulse I (102.62 °/s).

**Figure 4:**
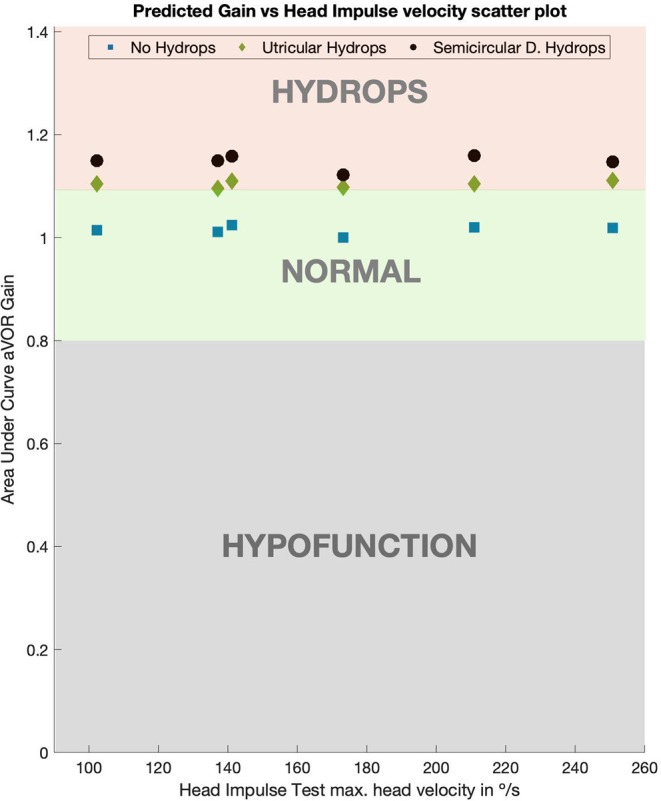
Predicted values of aVOR gain for normal, utricular and canal hydrops models. For both (utricular and canal) hydrops models aVOR gains were always >1.09 on all the tested head impulse velocities. Gains for the utricular model were slightly lower than for the canal model, but always over 0.1 more than the normal model gain values, which had gain value of 1.

The computed aVOR gain values using area under the curve method (Table 2, Figure 4) did show a mean aVOR gain of 1.01 ± 0.008 for the no-hydrops model, a mean gain of 1.14 ± 0.014 for the canal hydrops model, and a mean gain of 1.10 ± 0.007 for the utricular hydrops model.

**Table 2:** Gain (aVOR) values computed using area-under-curve method for predicted eye velocity values and real measured head velocity values for the six head impulse tests included and the three hydrops models included in this study.

**Impulse**	**Hydrops Model**	**Predicted aVOR gain**
I	No Hydrops	1.014
II	No Hydrops	1.010
III	No Hydrops	1.024
IV	No Hydrops	1.000
V	No Hydrops	1.019
VI	No Hydrops	1.019
I	Canal Hydrops	1.149
II	Canal Hydrops	1.148
III	Canal Hydrops	1.158
IV	Canal Hydrops	1.122
V	Canal Hydrops	1.159
VI	Canal Hydrops	1.149
I	Utricular Hydrops	1.104
II	Utricular Hydrops	1.095
III	Utricular Hydrops	1.109
IV	Utricular Hydrops	1.091
V	Utricular Hydrops	1.104
VI	Utricular Hydrops	1.111

## Discussion

In this study we predicted the endolymph hydrodynamics in geometric models of both normal and hydropic vestibular membranous labyrinths, using computer fluid dynamics software during the movement condition of the head impulse test.

Probably the most important question before discussing the computations performed in this research itself is about the actual evidence of the presence of vestibular hydrops affecting utriculus and semicircular canals in Menière disease and near spectrum human pathologies.

Although there are published cases of utricular and canal hydrops observation on 3 Tesla magnetic resonance imaging (MRI) with specific endolymph sequences (17), most actual imaging sequences of Menière disease and endolymphatic hydrops show cochlear and saccular hydrops, but not utricular or canal hydrops, which are rare findings in the published literature. It is not clear if this absence of MRI hydrops is related to a real lack of hydrops in these structures or other factors could be underlie these “anecdotal” cases of hydrops location. Probably, as suggested by Lopez-Escamez on a recent systematic review of the published papers on hydrops detection on 3 Tesla MRI sequences, actual MRI methods to adequate evaluate the endolymphatic hydrops are too novel and heterogenous to be considered as mature and reproducible techniques, especially in patients with early Menière disease (18).

On the other hand, despite the fact that most histologic published papers are focused on cochlear hydrops there are some histologic studies that strongly support the idea that utricular and canal hydrops may be also (relatively) frequent in the Menière disease population. Okuno and Sando (19) described the histopathological findings in 22 temporal bones from Menière's disease patients. Regarding vestibular organ hydrops, these authors found that saccular hydrops was present in 19 of 22 (86%) temporal bones, but also 11 of 22 (50%) temporal bones showed evident hydrops signals in the utricle region and 5–8 (23–36%) showed hydrops on at least one of the semicircular canals, the posterior canal being the most often affected (8/22) 36%. Concerning the severity of hydrops, these authors also described that saccular hydrops is less conservative with the original vestibular organ anatomy than utricular and semicircular canal hydrops are. Considering the histologic evidences published by Okuno and Sando, utricular hydrops could not be considered as infrequent, because it was present in 50% of temporal bones; also, canal hydrops should not be considered as a rare event, with a 23–35% of prevalence on temporal bones from Menière's disease patients.

The lack of an actual descriptive geometric model of the vestibular organ hydrops developed from radiological or histological findings forced us to develop a hydrops models based on McGarvie (11) descriptions but also inside the real measurements published by Okundo and Sando (19). Unfortunately, at the present moment, we were not able to find a more precise source to develop our vestibular hydrops models. This limitation in our models could be solved with future histological and radiological data or performing new CFD simulations, based on the here presented, where the hydrops geometry will be the main parameter to be tested.

In the developed models we only considered one volumetric dilatation ratio for each model. The effect of other dilatation ratios and its influence on VOR gain values should be evaluated on further studies, this will be of particular interest to determine how will be the eye velocity enhancement at different Menière disease clinical stages.

Obviously, despite computer simulations being plausible from a physical point of view, they are not the strongest scientific evidence and also have limitations. In our computations we used anatomic 3D models from microCT reconstruction (Figure 1B), that have never before been used with this purpose in vestibular physiopathology research. In our model we considered the membranous labyrinth to consist of rigid walls, when really, they are elastic, and this elasticity could influence the endolymph flow during the head impulse test (20).

In our model cupula region was represented as a rigid wall. Goyens et al. (21) has recently evaluated the effect of different cupula designs and its biophysical effects on main variables that could affect to the VOR responses, concluding that the use of simplified models where the cupula is represented as a rigid wall, the same as we used in our model, are also adequate and valid models to evaluate the vestibular system biomechanics. Also, other vestibular models, like the model developed by Grieser et al. (6) that includes an elastic cupula in a simplified vestibular model has outputted similar enhancement of eye velocity responses reported by here.

The evidence of the concordance between models supports the mathematical approach used in our model to predict the (hypothetical) cupula deflection from a given variation of transcupular pressure over time which appears to be an acceptable approximation to overcome the lack of an anatomical and elastic cupula in our model. Other limitation about this research is that we build a model where only the information of ipsilateral afference vestibular signal was computed and the contralateral vestibular organ was not included on our model. To reach a better understanding of hydrops effect on vestibular system a more complete vestibular model, including contralateral vestibular organ signal should be considered on further researches.

Also, although microCT reconstruction of the vestibular labyrinth has a high resolution, the anatomic details of the ampullar receptor were not included in our models, and the cupula region was modeled as a simple wall, not including other geometric and elastic details. With the obtained computations we suggest that micro-CT geometric models using CFD software to predict endolymph flow appear to be a very strong experimental method that could be applied to other vestibular function tests and other models of vestibular pathology.

As similar experimental research that partially supports the results obtained in this study, Yamauchi et al. (22) found that in animal models of horizontal canal hydrops that afferent vestibular nerve discharge is increased by the experimental induction of a hydropic environment, for both during static conditions and during sinusoidal (not impulsive) head movements. Nevertheless, Yamauchi et al. (22) describes that the static increase of discharge is adapted by the system along time and the dynamic (sinusoidal) response is variable depending on stimulus frequency. A very interesting point in this research is that Yamauchi et al. also describes that the increased vestibular discharges observed due the hydrops are not sustained over time. To explain this, the authors suggests that the vestibular system adaptation circuits will decrease these increased responses along time. If this vestibular adaptation mechanism is also applied to our human vestibular model it is probable that the enhanced eye responses will be more evident on the nearest time to the hydrops attack and probably will be decreased (due vestibular system adaptation) over time.

In this research results, we found that for head impulses of all head velocities, the velocities of the eye response are amplified by a 1.14 factor; for the canal hydrops model this was also in concordance with the aVOR gain values obtained in the canal hydrops model (Figure 4). With a slightly lower magnitude, aVOR gain was also significantly enhanced in the utricular hydrops model. These predicted enhanced eye velocities and aVOR gains were observed in the same proportion on all the head impulses included in this study. Because we obtained a 1.01 mean value for aVOR gain on non-hydrops models, the gain enhancement predicted by this study can be only directly extrapolated to right-side head impulse tests, in which the vHIT camera records responses of the right eye. This is because a gain value of 1 is only observed on the right side impulses of vHIT device with right-side mounted camera, and left-side impulses have a slightly lower than 1 aVOR gain value (23). But probably x1.14 enhancement of velocity data could be applied to left-side impulses.

Histopathological findings have also pointed a possible vestibular labyrinth structural alteration that, owing to the effect of hydrops, could affect to the pressure on ampullar receptors of horizontal semicircular canal. In the temporal bone series presented by Okuno and Sando (19) severe saccular hydrops had caused in 7 of 22 (31%) temporal bones a herniation of part of the saccular dilated structure into non-ampullar side of the horizontal side of this semicircular canal. Although our models did not compute the effect on ampullar pressure of this saccular herniation into the posterior region of the horizontal canal, it is probable that this herniation will also provoke an enhanced eye velocity response, probably greater than the observed in our models.

From a more hypothetical point of view, but based on findings of this study and the actual evidence of utricular or semicircular hydrops incidence in Menière disease patients (17–19), the enhanced eye velocity sign should appear in no more than half of Menière's disease patients, according to the described prevalence of canal and utricular hydrops (19). But there is a second factor that should be considered in addition to hydrops location: the time of evolution of the Menière disease. It has been described that for longstanding Menière disease patients type II hair cell vestibular receptors number is decreased and also an even more significant loss of cells on Scarpa's ganglion (24). These two histopathological findings present in patient with well-defined Menière disease could predict that the (related to hydrops) enhanced eye velocity will decrease to a normal or reduced eye velocity response due to the loss of sensitivity of vestibular receptors. Because this cellular damage depends on the time of evolution of the Menière disease, the initial prevalence of enhanced eye responses will also decrease with time. Considering these factors, it is probable that enhanced eye velocities will be only observed only in a fraction of Menière disease patients, and should be more prevalent in patients in the (relatively) early stage of the disease.

This theoretical prevalence of enhanced eye velocity responses in early Menière patients could suggest that enhanced velocity on head impulse test will be a very specific sign for early stages of Menière disease, but with a limited clinical sensibility due to the relatively low incidence of utricular or semicircular hydrops.

We conclude that, with the described limitations, the results of our physical computation model support the hypothesis that in endolymphatic hydrops conditions, when they are affecting horizontal semicircular canal and utricular region on moderate dilatations, the eye velocity responses output by the aVOR will be enhanced by an 1.14 factor, and aVOR gain values will be over 1.1 for right-sided impulses. These predictions must be confirmed by clinical trials, but should be considered in clinical practice, especially when cases of early Menière disease are suspected.

## Data Availability Statement

All datasets generated for this study are included in the article/Supplementary Material.

## Ethics Statement

This study was designed and performed in accordance with the ethical guidelines of the 1975 Declaration of Helsinki.

## Author Contributions

JR-M designed the computation model, performed the computer simulations and redacted the manuscript. XA co-designed the computation model and redacted the manuscript. KC developed the 3D geometric model and redacted the manuscript. AB submitted the clinical data, reviewed and redacted the manuscript. IC co-designed the computation model, redacted the manuscript and reviewed and supervised this research.

## Conflict of Interest

The authors declare that the research was conducted in the absence of any commercial or financial relationships that could be construed as a potential conflict of interest.

## Supplementary Material

Appendix Videos 1–3 Paraview software exported videos of the normal (Video S1), canal hydrops (Video S2) and utricular hydrops (Video S3) models for the raw head impulse test VI.

Appendix Figure 1 Video head impulse tests used in the simulations. Traces of head velocity versus time for the set 6 head impulses, obtained from a real vHIT measurement database after lowpass filter postprocess, with peak head velocity varying in approximately equal steps from 102.62 °/s to 249.47 °/s.

Appendix Figure 2 Increase of ampullar pressure during the head impulse test for the six progressive velocity head impulses computed on the three hydrops models. Higher pressure values were measured in both the models of canal hydrops, and those of utricular hydrops.

Appendix Figure 3 Predicted eye velocity values during head impulse test for the six progressive velocity head impulses computed on the four hydrops models, showing the pressure values measured, assuming semicircular receptors to be highly overdamped accelerometer sensors. Higher velocity values were measured in both the models of canal hydrops, and those of utricular hydrops.

Appendix Simulation Files (Datasheet 1–5) Compressed in this folder the reader can find: The anatomic 3D models folder, where the STL files used in this research can be found, these models are also automatically incorporated to the simulations folders. The head impulses folder, where the CSV files containing the head velocity real data used in this research can be found, these files are ready to be imported to the simulation software. In the “Dynamic mesh” section of the software, reader should select rigid type and rotating motion with omega from table, selecting the impulse of interest to be used as simulated head impulse and also setting the origin on movement to a medial point of the utricular region. In the Hydrops_SC, Hydrops_UC and NoHydrops folders configuration files ready to be open by the SimFlow 3.1 version simulation software are provided for each 3D STL model of the vestibular labyrinth. See methods section for detailed info about simulation software reference.
