# Supplementary figures and images for: Computing Endolymph Hydrodynamics During Head Impulse Test on Normal and Hydropic Vestibular Labyrinth Models
Source: Front Neurol. 2020 Apr 21;11:289. doi: 10.3389/fneur.2020.00289 (PMC7193182; doi:10.3389/fneur.2020.00289)

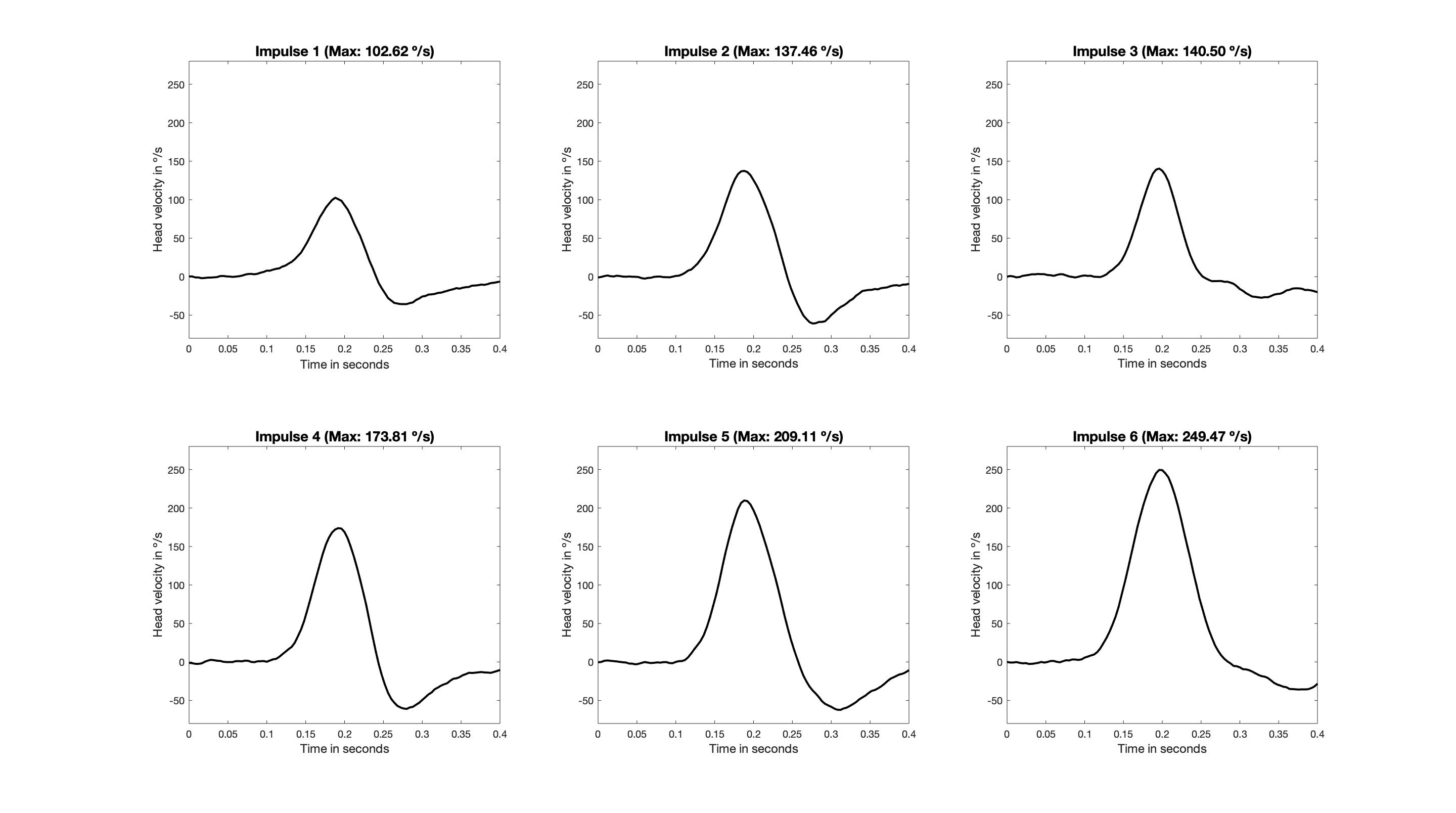

Supplement: Appendix Figure 1 — Video head impulse tests used in the simulations. Traces of head velocity versus time for the set 6 head impulses, obtained from a real vHIT measurement database after lowpass filter postprocess, with peak head velocity varying in approximately equal steps from 102.62 °/s to 249.47 °/s. [file Image_1.JPEG]

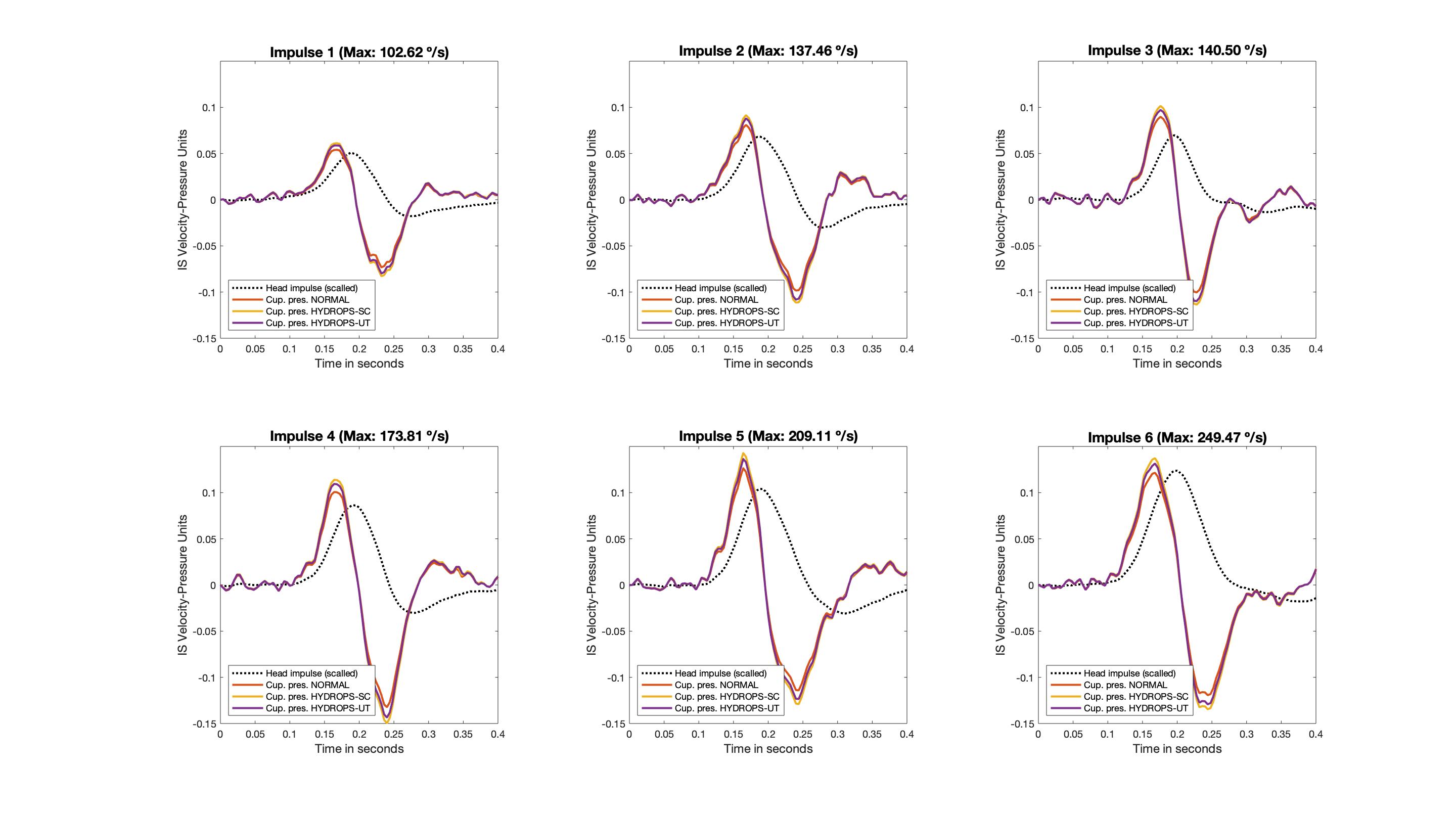

Supplement: Appendix Figure 2 — Increase of ampullar pressure during the head impulse test for the six progressive velocity head impulses computed on the three hydrops models. Higher pressure values were measured in both the models of canal hydrops, and those of utricular hydrops. [file Image_2.JPEG]

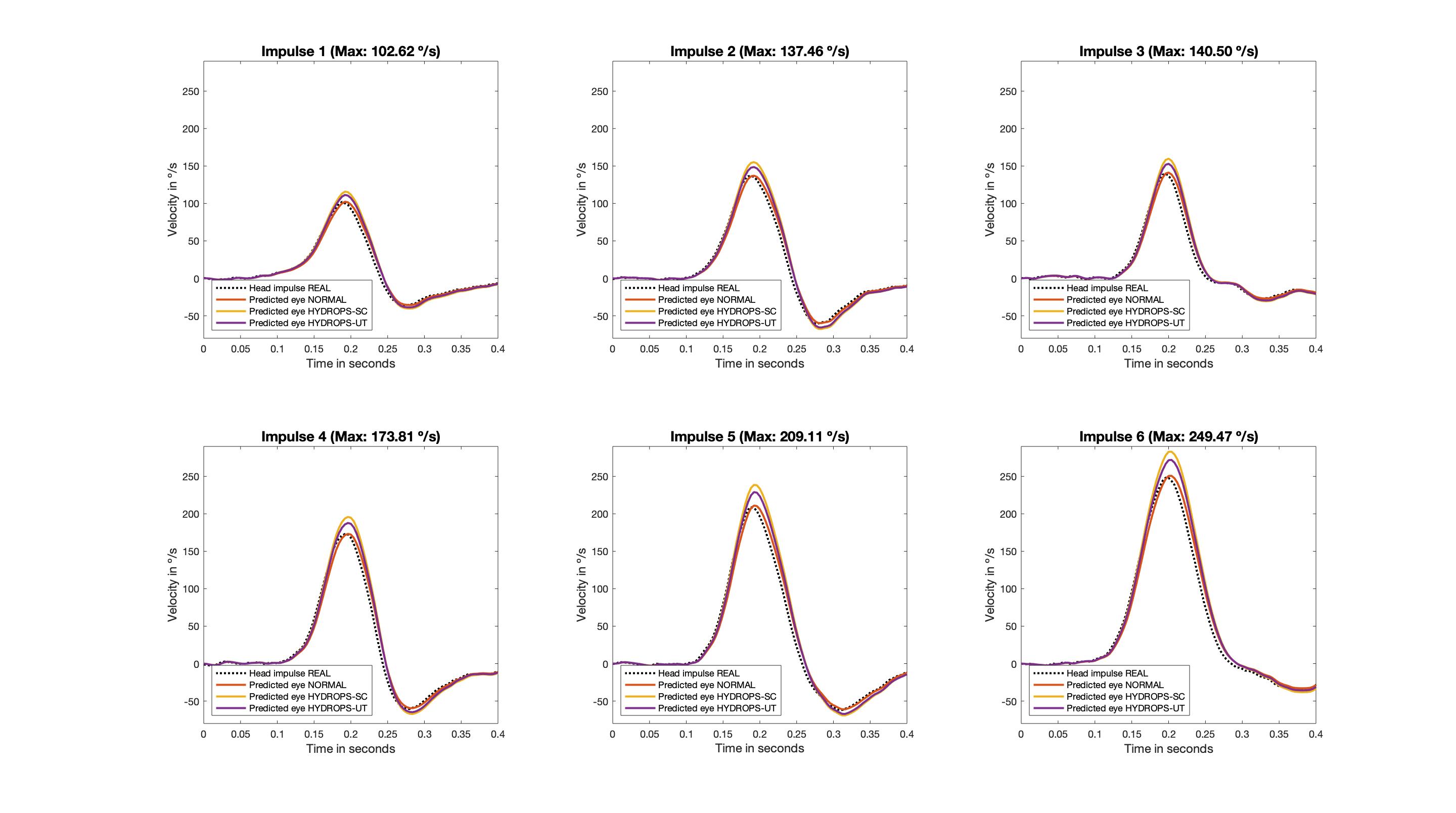

Supplement: Appendix Figure 3 — Predicted eye velocity values during head impulse test for the six progressive velocity head impulses computed on the four hydrops models, showing the pressure values measured, assuming semicircular receptors to be highly overdamped accelerometer sensors. Higher velocity values were measured in both the models of canal hydrops, and those of utricular hydrops. [file Image_3.JPEG]
